# The role of orexin in post-stroke inflammation, cognitive decline, and depression

**DOI:** 10.1186/s13041-015-0106-1

**Published:** 2015-03-11

**Authors:** Juhyun Song, Eosu Kim, Chul-Hoon Kim, Ho-Taek Song, Jong Eun Lee

**Affiliations:** Department of Anatomy, Yonsei University College of Medicine, Seoul, 120-752 South Korea; BK21 Plus Project for Medical Sciences, and Brain Research Institute, Yonsei University, College of Medicine, Seoul, 120-752 South Korea; Department of Psychiatry, Yonsei University College of Medicine, 120-752 Seoul, South Korea; Department of Pharmacology, Yonsei University College of Medicine, 120-752 Seoul, South Korea; Department of Diagnostic Radiology, Yonsei University College of Medicine, 120-752 Seoul, South Korea

**Keywords:** Post-stroke, Orexin, Inflammation, Cognitive dysfunction, Depression

## Abstract

Ischemic stroke results in diverse pathophysiologies, including cerebral inflammation, neuronal loss, cognitive dysfunction, and depression. Studies aimed at identifying therapeutic solutions to alleviate these outcomes are important due to the increase in the number of stroke patients annually. Recently, many studies have reported that orexin, commonly known as a neuropeptide regulator of sleep/wakefulness and appetite, is associated with neuronal cell apoptosis, memory function, and depressive symptoms. Here, we briefly summarize recent studies regarding the role and future perspectives of orexin in post-ischemic stroke. This review advances our understanding of the role of orexin in post-stroke pathologies, focusing on its possible function as a therapeutic regulator in the post-ischemic brain. Ultimately, we suggest the clinical potential of orexin to regulate post-stroke pathologies.

## Introduction

Ischemia resulting from a disturbance of cerebral blood flow is one of the leading causes of morbidity and mortality worldwide, resulting in permanent disability [1,2]. The prevalence of stroke was estimated almost 5.7 million people in 2005 [2,3] and was expected the increase of prevalence in the future according to global researches [4-6]. Stroke is related to several diseases, including hypertension, dyslipidemia, and obesity [7,8]. Recently, stroke has emerged as a direct cause of dementia [9]. Some stroke patients are diagnosed with dementia or show cognitive decline [10]. Additionally, new-onset dementia occurs in 5.4% of patients older than 60 years and 10.4% of patients older than 90 years 1 year after a stroke [11]. Furthermore, several studies indicate a strong relationship between stroke and depression [12-14]. Several studies also report that the prevalence of post-stroke depression is more than 22.5% [13,14]. Orexin peptides (orexin-A and orexin-B) produced by the lateral hypothalamus are known to regulate feeding, energy homeostasis, neuroendocrine activities, and the sleep-wake cycle by binding to orexin-1 (OX1R) and orexin-2 (OX2R) receptors [15-18]. OX1R is commonly present in the tenia tecta, dorsal raphe nucleus and Cornu Ammonis (CA)1, CA2, indusium griseum, septohippocampal nucleus in brain [19,20]. OX2R is abundant in brain regions related to basal ganglia such as the ventral striatum and subthalamic nucleus [19,21-23]. Some studies have elucidated the role of orexin in blood pressure regulation [24], inflammation [25], memory function [26,27], and depression [28]. In this review, we highlight recent studies regarding the role of orexin in the brain following ischemic stroke, particularly emphasizing studies on the role of orexin in inflammation, cognitive dysfunction, and depression following post-ischemic stroke.

### Post-ischemic stroke

#### Inflammation following a stroke

Ischemic stroke results in inflammation in the brain, which can directly influence the repair of neural damage and subsequent pathologies [29]. Inflammation is commonly regarded as necessary for the clearance of the large amount of debris caused by brain cell necrotic death [30,31]. After a stroke, cerebral inflammation exacerbates vascular dysfunction and leads to severe neuronal cell death [32]. Post-ischemic inflammation, which is a crucial process in the pathophysiology of ischemic stroke, is associated with post-stroke prognosis [29,30,32-35]. The distinct features of ischemic stroke are not only a large amount of necrotic neuronal death but also extreme infiltration of immune cells [29,30]. After the mild middle cerebral artery occlusion, cytochrome-C release and caspase processing are observed at 6 and 9 hour, and cell death are reported the severe inflammation of post-ischemic stroke between 24 and 72 hour [29,32]. Severe inflammation results in secondary brain damage [36] such as cerebral swelling (i.e., brain edema), which is often fatal in ischemic stroke patients [29,30]. Modulation of the inflammatory response after a stroke is important due to the direct association between inflammation and secondary damage following a stroke.

### Cognitive impairment following a stroke

According to recent studies, stroke is an emerging risk factor for dementia [37]. A previous study identified dementia after a stroke as vascular dementia [38]. However, a recent study used the term post-stroke dementia (PSD) to define any dementia occurring after a stroke [39]. PSD includes all dementias occurring after a stroke, including vascular dementia, Alzheimer’s disease (AD), and mixed dementia (vascular dementia with AD) [39,40]. Dementia is associated with neuronal dysfunction and neuronal death, causing cognitive impairment [41]. Approximately 30% of stroke patients suffer cognitive impairment after a stroke [42] and develop dementia within 1 year of stroke onset [43]. Some clinical studies also report the presence of AD-related pathogenesis in one-third of dementia cases after a stroke [44]. According to recent studies, a high proportion of stroke patients exhibit cognitive impairment within 3 months after a stroke [45], and 47.3% of first-stroke patients show memory loss 3 month after a stroke [46]. Therefore, several studies have continued to search for therapeutic solutions for PSD. One study utilizing a middle cerebral artery occlusion (MCAO) animal model suggests that reduced activity of extracellular regulated protein kinase (ERK) in the bilateral hippocampi may contribute to cognitive impairment after ischemic stroke [47]. Another study focused on a novel neurotransmitter that could decrease hippocampal neuronal damage and thereby alleviate cognitive impairment after ischemic stroke [48].

### Depression

Depression following ischemic stroke is termed post-stroke depression and is considered the most frequently observed psychiatric problem after cerebral ischemia [49]. A recent study reports that the prevalence of depression after a stroke ranges from 39% to 52% within 5 years following a stroke [12]. Post-stroke depression commonly occurs approximately 2 to 3 years following a stroke [50]. In the 1970s, the identification of depression following a stroke led to the concept that clinical depression after a stroke could be a consequence of brain damage [51,52]. A recent study reports that 33% of all stroke survivors show depressive symptoms based on research conducted between 1977 and 2002 [53]. In addition, clinical studies report that post-stroke depression could affect the recovery of function and cognitive ability [54-56]. One study reports that stroke patients who show improvements in cognitive function in the 3 months following the onset of a stroke show greater improvements in their levels of depression [57]. Post-stroke depression is an important issue that it is linked to the progression of other stroke pathologies and could affect functional recovery after a stroke.

### Orexin

The orexins, named for the Greek word for appetite [58], stimulate appetite [58] and are the common name given to the neuropeptide and the neurotransmitter [21] called orexin-A and orexin-B (also known as hypocretin-1 [HCRT-1] and hypocretin-2 [HCRT-2]) [58,59]. Orexins work by activating two G-protein-coupled receptors that are differentially expressed throughout the brain [19,20], orexin receptor 1 (OXR1) and orexin receptor 2 (OXR2) (also named HCRTR1 and HCRTR2 [60]). Orexin-A has equal affinity for both OXR1 and OXR2 receptors, whereas orexin-B acts primarily on OX2R [21,61,62]. The activation of OX2R by orexin-A or -B opens nonselective cation channels to depolarize orexin neurons [63], and regulates the opening of K channels [64,65], and promotes the release of presynaptic glutamate [63,66], and gamma-aminobutyric acid (GABA) [64]. Particularly, orexin-A rapidly crosses the blood–brain barrier (BBB) [67]. Orexins are produced by neurons mainly located in the lateral hypothalamic area [58,68]. These neurons send widespread projections into the prefrontal cortex, hippocampus, thalamus, and hypothalamus [69]. Orexin neurons also play crucial roles in the regulation of sleep and wakefulness [58,70,71], appetite [72-74], and energy homeostasis [75]. Orexin neurons detect nutritional status by reacting to peripheral metabolic signals such as glucose and appetite-related hormones (leptin and ghrelin) [76-78] and controlling the production glucose [79-81] and vital gases [82], and also receive the various neural signal inputs [83]. Moreover, orexin projected to cardiovascular regulatory centers in the hindbrain [21], and projected to the areas including locus coeruleus, raphe nuclei, parabrachial nuclei, central gray and nucleus tractus solitarius [69] which regulate peripheral blood pressure [69,84,85]. Several studies also show the cardiovascular effect of orexin through intracisternal and intrathecal injections of orexin-A and -B in the vasopressor area of the brain [86,87]. One study reports that orexin knock-out mice exhibit a reduced basal blood pressure response to motivated behavior [88]. In addition, the orexinergic system plays important roles in the regulation of depression-related neurophysiological processes, including cognitive processes [89,90]. The projection of orexinergic neurons to the hippocampus is implicated in learning and memory function [26,27,91]. Recent studies show that the nasal administration of orexin-A alleviates cognitive impairment in orexin/ataxin-3-transgenic mice [92]. Another clinical study demonstrates that lower orexin-A levels in cerebrospinal fluid are involved in learning and memory impairments caused by epilepsy [93]. Orexin levels in plasma and hypothalamus in brain were reduced in animal study [94] and orexin’s concentration in serum and cerebrospinal fluid (CSF) also were low level in stroke patients [95]. Considering the results of these previous studies, orexins may play multiple roles by binding orexin receptors in diverse pathophysiologies after a stroke and the development of brain injury.

### The role of orexin in post-ischemic stroke

#### The relationship between orexin and risk factors for a stroke

Orexin is involved in blood pressure regulation [96,97]. Orexin knock-out mice and orexin neuron-ablated transgenic rats have lower basal blood pressure [24,98]. Additionally, the orexin system participates in the pathogenesis of high blood pressure in spontaneously hypertensive rats [24,99]. One study reported that the blockage of orexin receptors attenuates blood pressure in hypertensive rats [97]. In another *in vivo* study, the central administration of orexin in animals increases arterial blood pressure and heart rate, and these effects are attenuated by treatment with orexin receptor antagonists [100-104]. Intracerebroventricular injection of orexin-A increases arterial pressure in rats and rabbits [85,105]. One study demonstrated that intravenous administration of orexin decreases infarct volume by increasing cerebral blood flow [106]. Acute intracerebroventricular injection of orexin-B also increases arterial pressure [85]. Given that blood pressure is a risk factor for a stroke [107-109], the promotion of orexin secretion may be involved in the onset of stroke by regulating blood pressure.

### The role of orexin in inflammation after a stroke

Several studies demonstrate that orexin-A inhibits apoptosis and lipid peroxidation in a hypothalamic cell model [25,106,110]. Another study reports that inflammation conditions induced by lipopolysaccharide administration lead to orexin neuron damage [111]. Recent studies highlighted the anti-inflammatory function of orexin in neuroinflammation diseases [112]and oxidative stress caused by cerebral ischemia [113]. In addition, orexin-A mRNA level is decreased under acute inflammation conditions [114]. The cellular response to orexin receptor activation is increased intracellular Ca^2+^ influx by protein kinase C-dependent activation or voltage-gated Ca^2+^ receptors [115,116]. The common downstream pathways of activated orexin receptors involve the activity of extracellular-signal-regulated kinases (ERK1/2) and p38 mitogen-activated phosphate kinase (MAPK) [116,117]. Tumor necrosis factor alpha (TNF-α), a proinflammatory cytokine, impairs the function of the orexin system by decreasing levels of both prepro-hypocretin and OXR2 [118]. Moreover, intracerebroventricular administration of orexin-A before MCAO in rats [119,120] and mice [110] reduces infarct size. Orexin-A alters intracellular metabolic function and cell survival in neuronal tissue and cells [110,120-122]. Recent studies show that orexin-A exerts neuroprotective effects, including the activation of hypoxia-inducible factor-1α (HIF-1α) and reduction of oxidative stress [110,120]. Orexin-A increases ATP via induction of the transcription factor HIF-1α in mouse hypothalamic tissue under normoxic conditions [121]. Under ischemic conditions, orexin-A promotes the survival of primary cortical neurons *in vitro* and alleviates neuronal damage by modulating post-ischemic glucose intolerance *in vivo* [110,121]. In several clinical studies, a direct association between immunological problems [123-125] and orexin cell loss [126,127] is found in some narcolepsy patients. Indeed, narcolepsy patients exhibit elevated levels of TNF-α, interleukin (IL)-6, and p75 in their blood [128]. In addition, recent study reported that orexin-A regulates infection-induced inflammation by modulating the IL-6 and TNF-α in microglia and has protective role against ischemia stress [113]. Based on upper evidences, the elevation of orexin production may attenuate inflammation after a stroke and reduces the infarct size in brain.

### The role of orexin in cognitive impairment following a stroke

Orexins play a positive role in learning and memory function, suggesting that they are directly associated with the arousal process [26,129]. Orexin and its receptors (OX1R and OX2R) are widely distributed throughout the brain and thereby regulate learning and memory functions [26,27,91]. Specifically, orexin-A enables the acquisition, consolidation, and retrieval of learning and memory in a passive avoidance task even in the presence of an over-production of beta amyloid [27,91,130]. To date, some studies indicate an emerging role of the orexin system in the avoidance test [27,91,130] and Morris water maze test [26,131]. In detail, the inhibition of hippocampal OX1R using OX1R antagonism occurs in a deficit in cognitive processes based on the results of morris water maze task [131]. Also, the role of orexin-A demonstrated the contribution in the memory processing thorough T-maze footshock avoidance test and step-down inhibitory avoidance [27]. Orexins could increase the release of corticotrophin-releasing hormone (CRH) as well as the circulating levels of adrenocorticotropic hormone and glucocorticoids in the bloodstream [132,133]. Consequently, orexins are considered crucial regulators of monoaminergic neurotransmission [75]. A recent study shows that the activation of orexin neurons disrupts sleep [134]. The lateral hypothalamus is the most extensively interconnected area of the hypothalamus, allowing it to control diverse autonomic and somatomotor functions. Several studies have revealed direct projections from the lateral hypothalamus to hypothalamic, cortical, and limbic areas [135,136]. These connections are considered to represent the anatomical connectivity that supports sleep-wake regulation [69,137], energy homeostasis, and cognitive functions [138,139]. The function of the lateral hypothalamus depends on the function of orexin neurons that produce orexin-A and -B [21]. In an AD animal study, orexin was confirmed to improve memory in mice overproducing amyloid beta [27]. Additionally, treatment with orexin-A and OX1R exerts a neuroprotective effect and improves learning and memory in epilepsy [140]. Hippocampal neurogenesis plays a cardinal role in learning and memory, and the proliferation of immature neurons is particularly important due to their contribution to cognition [141-143]. Orexin-A and its receptors participate in neuronal cell proliferation and developmental mechanisms [144]. Considering upper evidences, we assume that the increase of orexin may improve cognitive impairment following a stroke.

### The role of orexin in depression following a stroke

Depression has considerable implications for the quality of life of affected individuals and is one of the most important causes of early death worldwide [145,146]. Depression induces distinctive neuroanatomical changes, including reducing the volume of the hippocampus and prefrontal cortex, which are brain regions that are important for inhibiting the stress response and restricting depressive behavior [147], and enlarging the amygdala [147-151]. Depression results from changes in various biochemical factors, including stress hormones, cytokines, neurotrophic factors such as brain-derived neurotrophic factor (BDNF), and neuropeptides such as orexins [152]. In Parkinson’s disease patients with depression, levels of BDNF [153] and orexin [154] are down-regulated. Recently, studies have demonstrated that neuropeptidergic dysregulation plays an essential role in the onset of depressive symptoms [155,156]. Since orexin’s discovery in 1998, the neuropeptide has been emerging as a promising target against depression [21]. A clinical case regarding the dysregulation of orexin release in depression was reported in 2003 [28]. Some suicidal patients show lower levels of orexin A than normal individuals [157,158]. Activation of orexin receptors promotes intracellular calcium influx through various intracellular signaling cascades that induce long-term potentiation [149-160]. A relationship between orexinergic neurotransmission and depression has been reported in a genetic rat model of depression [161]. Using orexin receptor knock-out mice, OXR2 was shown to have anti-depressive properties [162]. Specifically, this study showed that mice with increased OXR2 mRNA levels exhibit relatively normal behavior, whereas OXR2 knock-out mice exhibit depressive behavior [162]. In addition, orexin promotes the expression of BDNF [160,163,164], which regulates neuronal plasticity and is reduced in the blood serum of depression patients [165,166]. Moreover, an increased concentration of inflammatory cytokines in the brain is the major cause of depression in humans and animals [167-169]. One study showed that the relationship between depression and inflammation is strongly associated with alternations of synaptic plasticity and the metabolism of neurotransmitters involved in mood regulation [170]. Considering these lines of evidence, orexin may be involved in the onset of depression after a stroke. Furthermore, given that orexin regulates the inflammatory response, orexin may attenuate depressive symptoms after a stroke by attenuating inflammation.

## Conclusions

**Figure 1:**
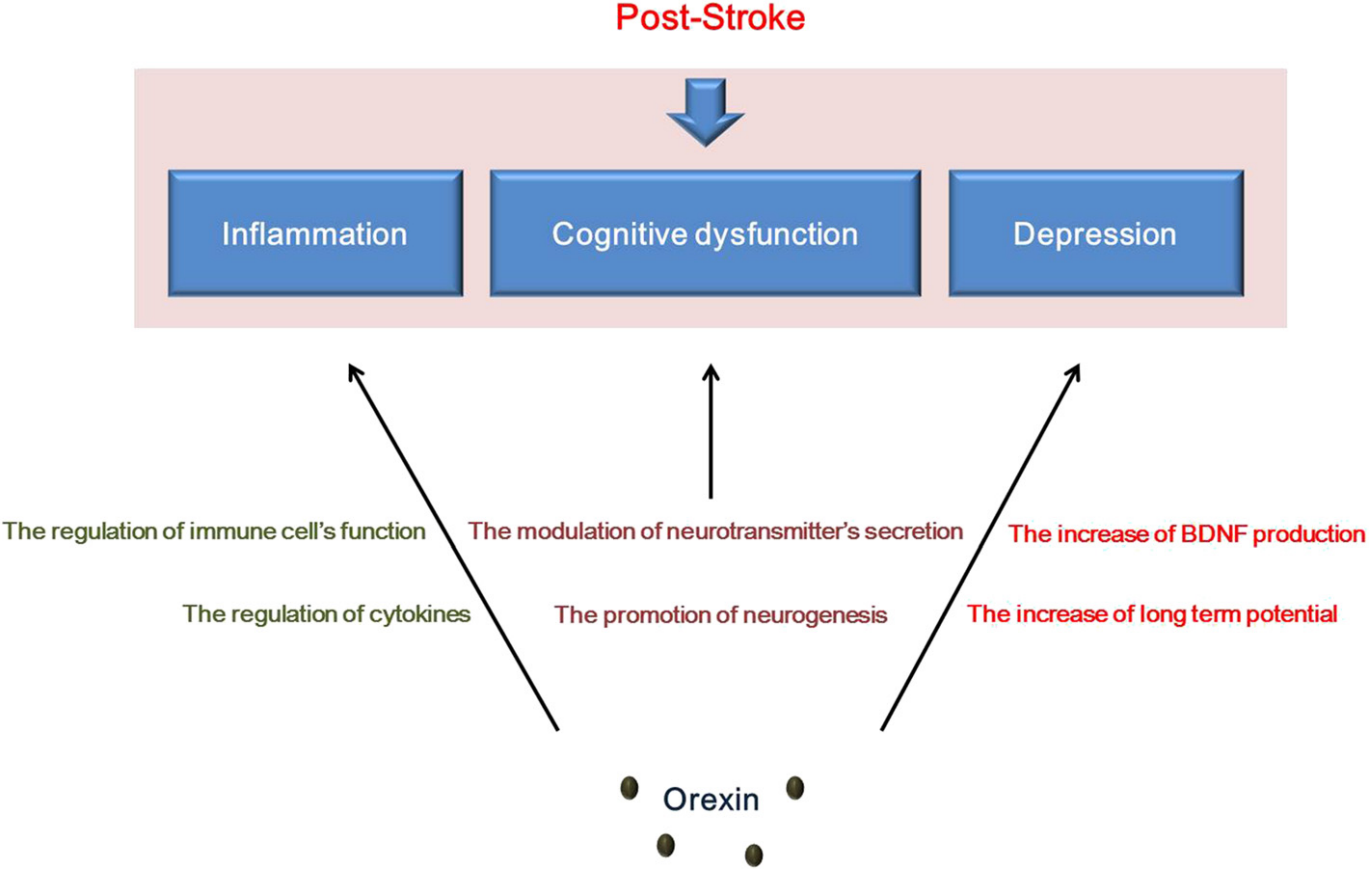
**The schematic image regarding the function of orexin in post** **-stroke.** This image presented that the function of orexin on pathogenesis after stroke such as inflammation, memory dysfunction, and depression. In inflammation caused by ischemic stress, orexin modulates the cytokine’s production to reduce the oxidative stress and stimulates the immune cells against post-stroke induced inflammation. In cognitive decline caused by stroke, orexin alleviates the learning impairment by regulating the secretion of neurotransmitters and also attenuates the memory loss by increasing the neurogenesis. In depression caused by stroke, orexin plays a beneficial role by accelerating the production of BDNF and facilitating the increase of long term potential.

Inflammation, cognitive impairment, and depression are distinctive features that appear after a stroke. To alleviate the pathophysiologies following a stroke, many researchers have studied the regulators of these phenomena. Orexin is a neuropeptide that is known to regulate appetite, metabolism, and sleep/awakeness. In this review, we focused on the emerging roles of orexin in post-stroke-related pathophysiologies. To conclude, this review highlights three remarkable roles of orexin after stroke: 1) orexin controls inflammation by regulating immune mediators such as pro-inflammatory cytokines after stroke; 2) orexin improves memory by modulating other neurotransmitters, and promoting hippocampal neurogenesis, and protecting the neuronal damage against post stroke induced oxidative stress; 3) orexin mitigates depression by accelerating neurotrophic factor secretion and by promoting long term potentiation through calcium influx’s increase (Figure 1). Although studies concerning the post-stroke role of orexin are still in preliminary stages, further studies involving the function of orexin after stroke might suggest the potential clinical value of orexin as an effective therapeutic modulator to alleviate pathologies following a stroke.
